# Hepatocellular Carcinoma in The Gambia and the role of Hepatitis B and Hepatitis C

**DOI:** 10.1186/1477-7800-2-20

**Published:** 2005-10-04

**Authors:** Clement Ibi Mboto, Angela Davies-Russell, Mark Fielder, Andrew Paul Jewell

**Affiliations:** 1 Royal Victoria Hospital, Banjul, The Gambia; 2 School of Life Science, Kingston University, Surrey KT1 2EE, UK; 3 Faculty of Health and Social Care Sciences, Kingston University and St George's University of London, Surrey KT1 2EE, UK

**Keywords:** Hepatitis B, hepatitis C, hepatocellular carcinoma, The Gambia

## Abstract

**Objectives:**

Hepatocellular Carcinoma is the commonest form of cancer in The Gambia, and although Hepatitis B and Hepatitis C are known risk factors, accurate baseline data on Hepatitis B and Hepatitis C distribution in the region are limited. Similarly data including information on the involvement of the viruses in HCC remains unknown. The current study was undertaken to estimate the risk of HCC in relation to HCV and HBV in The Gambia.

**Methods:**

Thirteen patients with histological proven history of HCC and 39 healthy controls were enrolled in the study. Each subject blood was screened individually for anti-HCV using ORTHO HCV 3.0 ELISA test system (Ortho-Clinical Diagnostics, Inc, U.S.A) and for HBsAg using QUADRATECH CHECK 4-HBs one step generation hepatitis B surface antigen test kit (VEDALAB, France) following the manufacturers instructions.

**Results:**

HBsAg and anti-HCV was detected in 38.5 %(5/13) and 7.7% (1/39) of the persons with a history of HCC respectively. HBsAg but not anti-HCV was detected in 12.8% (5/39 of the case control subjects. HBsAg and HCV rates among the HCC patients were higher in men than women. Rates were highest in patients 48 years and above (37.5%; 3/8). No patient was found with anti-HCV and anti-HBV.

**Conclusion:**

These results indicate that the involvement of HBV and HCV in HCC in the country is in a ratio of 5:1 and that these two viruses might be independently involved in the pathogenesis of the disease. The study revealed a statistically significant association (p = 0.04) between HBsAg and HCC patients.

The results also indicate that up to 50% of HCC cases in the country may be due to non viral factors and calls for further studies in this regard. These findings call for provision of diagnostic facilities for these viruses in hospitals and for their routine screening in blood banks while intervention programmes should be put in place.

## Introduction

Hepatocellular carcinoma (HCC) is a common cancer worldwide, which occurs substantially as a complication of liver cirrhosis [1]. Chronic infection with Hepatitis B virus (HBV) and Hepatitis C virus (HCV) has been associated with the disease [2,3] with higher incidences reported in countries where Hepatitis B and or Hepatitis C are endemic [4,5] HCV has a lower global prevalence than HBV and is more often associated with HCC in economically developed regions [6,7]. Globally, HCC is increasingly becoming a major health concern with estimates of 500,000 new cases reported annually [8]. Some studies have shown a direct correlation between the geographical distribution of HBV and HCV and HCC prevalence [7]. In Japan and Italy where higher HCV prevalence is very high, HCC has been shown to be more prevalent [8]. In the United States of America, it is estimated that the disease burden from HCV is likely to rise considerably over the next 10–20 years increasing demands on liver transplantation [9]. In West Africa, some studies have shown that both HBV and HCV infections are common but the role of HCV in acute infection is still not clear [10-13].

In the Gambia hepatocellular carcinoma (HCC) has been defined as the country's commonest form of cancer [14], and Hepatitis B virus (HBV) infection is endemic [15,16]. However, like other developing countries in the West African region, accurate data including information about incidence and prevalence of both HBV and HCV or the involvement of the viruses in HCC is lacking or limited [13]. The problem is further compounded by the non-existence of facilities for HCV or HBV diagnosis in established hospitals in the country making it difficult to have a baseline data on HBV or HCV distribution in the country. The few available studies however, have shown Hepatitis B prevalence in the Gambia to be quite high [15,16]. The Gambia National blood transfusion services (GAMBLOOD), which were recently put in place, are yet to commence screening of blood for HCV or HBV. The present study was carried out to compare the involvement of HBV and HCV in HCC in the country.

## Subjects, Materials and Methods

This study is part of an on-going study on HIV and HCV coinfection and was approved by the Department of State for Health. The study population consisted of a total of 13 HCC patients seen consecutively at the Royal Victoria Teaching Hospital (RVTH), Banjul between the months of July to December 2002. The patients were aged 32 years to 76 years and were made up of 11 men and 2 women. Patients were enrolled for the study following informed consent. Each patient was matched by three persons on the basis of age and sex. The primary choice of control group persons were blood donors, however due to the lack of female blood donors, the two female HCC patients were matched with two women attending antenatal clinic in their first trimester of pregnancy, and four other female patients with history of malaria. In all a total of thirty-nine healthy controls made up of 33 blood donors and 6 women were enrolled for the study. Both the HCC patients and control subjects were unaware of their HCV or HBV status prior to the commencement of the study.

Blood samples were collected from each participant and linked by name and code number. Samples were separated within 8 hours of collection, screened individually for anti-HCV using ORTHO HCV 3.0 ELISA test system (Ortho-Clinical Diagnostics, Inc, U.S.A) a third generation enzyme linked immunosorbent assay (ELISA). Persons reactive to ORTHO HCV 3.0 ELISA test were considered anti-HCV positive [17]. Hepatitis B surface antigen (HBsAg) test was carried using QUADRATECH CHECK 4-HBs one-step generation hepatitis B surface antigen test kit (VEDALAB, France) following the manufacturers instructions.

HBsAg and anti-HCV prevalence rates were calculated to reflect the relative frequency of each disease while Odds ratio (OR) and ninety five percent confidence interval (95% CI) was calculated using the Fisher Exact Test to estimate the strength of the association between each infection and possible risk factor[18].

## Results

The mean age of the HCC patients was 46 years and 43 years for the men and women respectively. The mean age of the control subjects was 45.7 years for the men and 43 years for the women. The mean age of the HCC patients with HbsAg was 47.8 years, while the only patient with anti-HCV was aged 54 years.

Hepatitis B surface antigen (HbsAg) was present in 38.5 %(5/13) of the HCC patients (p = 0.04; 95% CI: 1.03–8.73) and in 12.8% (5/39) of the control subjects (p = 0.046; 95% CI: 0.11–0.97). Anti-HCV antibodies were detected in 7.7 % (1/13) of the HCC patients. No anti-HCV antibody was detected among any of the control subjects. Similarly no HBsAg or nor anti-HCV was detected in more than half (53%; 7/13) of the HCC patients.

The male HCC patients had an anti-HCV prevalence of 9.1 %(1/11) and a hepatitis B surface antigen (HBsAg) prevalence of 36.4 %(5/11). The two female HCC patients who participated in this study were both anti-HCV and HBsAg negative. No patient was found with anti-HCV and anti-HBV.

The hepatitis B surface antigen (HBsAg) prevalence for the control group was 12.12% (4/33) for the males (95% CI: 0.097–5.43; OR 0.69). The female control subjects had an HBsAg prevalence of 16.7 % (1/6) (CI: 0.18–10.27; OR: 1.45). All the HBsAg positive control subjects were blood donors give an HBsAg prevalence of 15.2 % (5/33). None of the control subjects demonstrated antibody to HCV.

Hepatitis B surface antigen rates were highest in patients 48 years and above (37.5%, 3/8) (CI: 0.23–3.79; OR: 0.9). Control subjects aged 48 years and above had a lower HbsAg prevalence (11.1%, 2/18) than those less than 48 years (15.8 %, 3/19)}; however this was not statistically significant (p > 0.05). There was a marginal statistically significant association between HBsAg and HCC patients (p = 0.04; 95%CI: 1.03–8.73, OR: 4.25).

**Figure 1:**
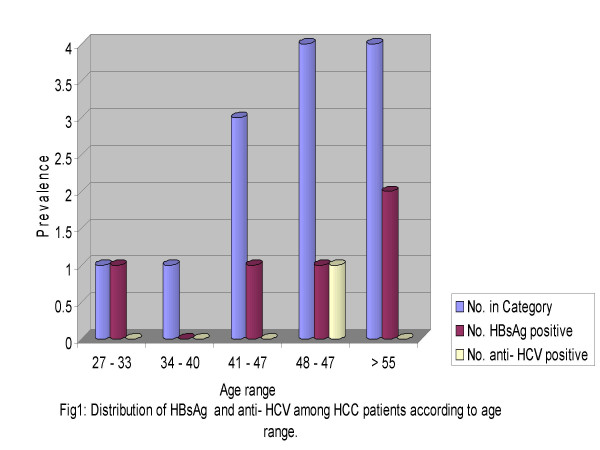
Distribution of HBsAg and anti-HCV among HCC patients according to age range.

A summary of the results of the hepatitis B surface antigen and anti-HCV tests for the HCC patients are shown in figure 1 below.

## Discussion

Hepatocellular carcinoma is generally associated with increasing age and significantly higher HBV and HCV prevalence have been reported among persons in their 40's and 50's respectively [4,19]. Some studies conducted in the West African region have found a comparatively higher HBsAg positivity in those 41 years and above.

In this study, patients had no prior knowledge of their HCV status because HCV testing was not performed routinely, and this test was not available before this study. A summary of the results showed that the mean age of the HCC patients with HbsAg as 48.2 years, while the only patient with anti-HCV was aged 54 years. This finding is in line with similar reports [4,19]

HCC is more associated with males than females [8]. The finding of a comparatively higher prevalence of HbsAg and HCV among the male subjects in this study reflects the results of other studies [8]. However, in this study the difference was not statistically significant (p > 0.05), although it may be due to the small number of women participants. A similar reason may be advanced for the finding of a comparatively higher prevalence of HBsAg among the female case control subjects than the males

This study reveals an HBsAg prevalence of 15.2 % (5/33) among the apparently healthy Gambian population. Even though the sample size in the study was small, the finding is of major public health significance. The finding also supports a report that suggests that the virus is endemic in the country [20]. An aggressive HBV immunization exercise carried out in the country is believed to have drastically reduced the incidence of HBV [14]. An earlier work almost a decade ago estimated an HBV prevalence of 15–20% among the Gambian population [20]. The prevalence found in this study may therefore be suggestive of stable existence of the virus in the region.

The finding of lower anti-HCV prevalence among the HCC patients and none among the control subjects reflects the lower prevalence of HCV and the possible low involvement in HCC in the country. These findings are in line with a recent report and also provide support for a similar work conducted in Senegal a country that shares an extensive border, language and cultural similarities with the Gambia [14,20]. Globally 52.3% of HCC is attributed to HBV while HCV account for about 25% [8].

The observation of higher rates of hepatitis B surface antigen (HBsAg), and anti-HCV in patients with HCC than in control subjects suggests the involvement of these viruses in HCC [21]. Similarly, the finding of anti-HCV and HBsAg independently among HCC patients suggests that these two viruses might contribute independently to the pathogenesis of HCC. This finding supports the assertion of the independent roles of HBV and HCV in the pathogenesis of HCC [5]. Some studies have reported the synergistic role of HBV and HCV in HCC [2]. In this study no patient was found with HBV and HCV infection simultaneously. This may however be due to the number of participants enrolled in the study.

The observed involvement of HBV and HCV in HCC patients in a ratio of 5:1 in this study is higher than that reported previously [14]. This difference could have resulted from the comparatively small study population. The World Health Organization estimated an HCV prevalence of 2.4% for West Africa region [22]. However, a close epidemiological association between the HCC and HCV was not found in Senegal [20]. Their findings suggest that the main viral cause of HCC in the Senegal remains HBV.

In this study the possible attributable fraction of HCC due to HBV or HCV is 46%, thus suggestive of the involvement of other factors. In some countries HCC has been associated with chronic exposure to toxins originating from *Aspergillus *infected grains and peanuts [8]. Other associated risk factors includes cigarette smoking, prolonged abuse of alcohol in addition to some hereditary factors [5,8,23]. These factors were not evaluated in this study nor are their contributory role as causative agents of HCC in the Gambia known, however grains are the country most staple food while cigarette smoking and groundnut consumption are very common habits in the Gambia. There is therefore need for studies to evaluate the possible involvement of non-viral factors in HCC in the country.

## Conclusion

These results suggest that HBV is endemic in the country and is present in apparently healthy persons. It also reveals that both HBV and HCV are actively involved in HCC in the region, in a ratio of 5:1 and that these two viruses might be independently involved in the pathogenesis of the disease. The results indicate that more than 50% of HCC cases in the country may be due to non-viral factors and calls for further studies to address this.

The study revealed a marginally statistically association (p = 0.04) between HBsAg and HCC patients (95%CI: 1.03–8.73, OR: 4.25). A similar level of association was found between HBsAg and with the case control subjects. No such association was found for HCV. These findings make it necessary for provision of diagnostic facilities for these viruses in hospitals and blood banks while intervention programmes should be put in place.

CIM designed the study and carried out laboratory work

AD analysed data and revised manuscript

MF critically revised manuscript

AJ conceived and organized the study, and revised the manuscript
